# Poor cardiovascular health is associated with subclinical atherosclerosis in apparently healthy sub-Saharan African populations: an H3Africa AWI-Gen study

**DOI:** 10.1186/s12916-021-01909-6

**Published:** 2021-02-10

**Authors:** Engelbert A. Nonterah, Nigel J. Crowther, Abraham Oduro, Godfred Agongo, Lisa K. Micklesfield, Palwendé R. Boua, Solomon S. R. Choma, Shukri F. Mohamed, Herman Sorgho, Stephen M. Tollman, Shane A. Norris, Frederick J. Raal, Diederick E. Grobbee, Michelé Ramsay, Michiel L. Bots, Kerstin Klipstein-Grobusch, as part of the H3Africa AWI-Gen study

**Affiliations:** 1 Clinical Sciences Department, Navrongo Health Research Centre, Ghana Health Service, Navrongo, Ghana; 2 Julius Global Health, Julius Center for Health Sciences and Primary Care, University Medical Center Utrecht, Utrecht University, Utrecht, the Netherlands; 3 Department of Chemical Pathology, National Health Laboratory Service, Faculty of Health Services, University of the Witwatersrand, Johannesburg, South Africa; 4 MRC/Wits Developmental Pathways for Health Research Unit, Faculty of Health Sciences, University of the Witwatersrand, Johannesburg, South Africa; 5 Institut de Recherché en Sciences de la Santé, Clinical Research Unit of Nanoro, Nanoro, Burkina Faso; 6 DIMAMO Health Demographic Surveillance Site, Department of Pathology and Medical Sciences, School of Health Care Sciences, Faculty of Health Sciences, University of Limpopo, Polokwane, South Africa; 7 African Population Health Research Centre, Nairobi, Kenya; 8 MRC/Wits Rural Public Health and Health Transitions Research Unit, School of Public Health, Faculty of Health Sciences, University of the Witwatersrand, Johannesburg, South Africa; 9 Carbohydrate and Lipid Metabolism Research Unit, Faculty of Health Sciences, University of the Witwatersrand, Johannesburg, South Africa; 10 Sydney Brenner Institute of Molecular Bioscience, Faculty of Health Sciences, University of the Witwatersrand, Johannesburg, South Africa; 11 Division of Epidemiology and Biostatistics, School of Public Health, Faculty of Health Sciences, University of the Witwatersrand, Johannesburg, South Africa

**Keywords:** Cardiovascular health index, Carotid intima-media thickness, Subclinical atherosclerosis, Sub-Saharan Africa, Primary prevention, Cardiovascular diseases, Screening, Understudied populations

## Abstract

**Background:**

The cardiovascular health index (CVHI) introduced by the American Heart Association is a valid, accessible, simple, and translatable metric for monitoring cardiovascular health in a population. Components of the CVHI include the following seven cardiovascular risk factors (often captured as life’s simple 7): smoking, dietary intake, physical activity, body mass index, blood pressure, glucose, and total cholesterol. We sought to expand the evidence for its utility to under-studied populations in sub-Saharan Africa, by determining its association with common carotid intima-media thickness (CIMT).

**Methods:**

We conducted a cross-sectional study involving 9011 participants drawn from Burkina Faso, Ghana, Kenya, and South Africa. We assessed established classical cardiovascular risk factors and measured carotid intima-media thickness of the left and right common carotid arteries using B-mode ultrasonography. Adjusted multilevel mixed-effect linear regression was used to determine the association of CVHI with common CIMT. In the combined population, an individual participant data meta-analyses random-effects was used to conduct pooled comparative sub-group analyses for differences between countries, sex, and socio-economic status.

**Results:**

The mean age of the study population was 51 ± 7 years and 51% were women, with a mean common CIMT of 637 ± 117 μm and CVHI score of 10.3 ± 2.0. Inverse associations were found between CVHI and common CIMT (β-coefficients [95% confidence interval]: Burkina Faso, − 6.51 [− 9.83, − 3.20] μm; Ghana, − 5.42 [− 8.90, − 1.95]; Kenya, − 6.58 [− 9.05, − 4.10]; and South Africa, − 7.85 [− 9.65, − 6.05]). Inverse relations were observed for women (− 4.44 [− 6.23, − 2.65]) and men (− 6.27 [− 7.91, − 4.64]) in the pooled sample. Smoking (*p* < 0.001), physical activity (*p* < 0.001), and hyperglycemia (*p* < 0.001) were related to CIMT in women only, while blood pressure and obesity were related to CIMT in both women and men (*p* < 0.001).

**Conclusion:**

This large pan-African population study demonstrates that CVHI is a strong marker of subclinical atherosclerosis, measured by common CIMT and importantly demonstrates that primary prevention of atherosclerotic cardiovascular disease in this understudied population should target physical activity, smoking, obesity, hypertension, and hyperglycemia.

**Supplementary Information:**

The online version contains supplementary material available at 10.1186/s12916-021-01909-6.

## Background

Cardiovascular disease (CVD) is a leading cause of global deaths [1, 2] raising huge public health concerns. Sub-Saharan Africa (SSA) is experiencing a rising burden of non-communicable diseases including CVD [2], alongside a reduction in the prevalence of infectious diseases [3]. Increasing urbanization, changing lifestyles, and population aging are probably accounting for the surge in the prevalence of CVD in SSA [3, 4]. This presents a challenge to the health systems of African countries and requires a pragmatic approach to the prevention of CVD. Where risk equations such as Framingham [5] and Pooled Cohort Equations (PCE) [6] have served as valuable tools in assessing the risk of CVD globally, these have inherent limitations especially when applied to an African population [7]. The American Heart Association and the American Stroke Association (AHA/ASA) developed the Cardiovascular Health Index (CVHI) as a new public health metric for assessing and monitoring cardiovascular health [8–11]. The components of the CVHI often referred to as life’s simple seven include smoking, dietary intake, physical activity, body mass index (BMI), blood pressure, glucose, and total cholesterol.

Due to the lack of data on incident CVD events, carotid-intima media thickness (CIMT) is often used as an alternate marker of CVD risk. Increased CIMT has been shown to be related to several risk factors for CVD and CVD risk globally [12, 13] including SSA populations [14].

The CVHI metric is proven to be related to CIMT, a marker of subclinical atherosclerosis [15], and coronary artery calcification [16] in both Caucasian and Asian populations [17]. In Africa, the association of CVHI with subclinical atherosclerosis has only been reported in a relatively small study of HIV-positive (*n* = 105) and non-infected (*n* = 100) individuals [18] in Uganda and recently in a rural setting in Limpopo, South Africa [19]. Expanding the evidence to a larger pan-African population would help to clarify whether the CVHI can be used as a simple screening tool for cardiovascular health within the existing health systems of resource-constrained SSA countries. Therefore, we set out to assess the association between cardiovascular health, using CVHI, and subclinical atherosclerosis in a large understudied African population living in SSA.

## Methods

### Study design and population

The H3Africa Africa-Wits-INDEPTH (www.indepthnetwork.org) partnership for genomic studies (AWI-Gen) project is a population-based cross-sectional study. Participants included older adult women and men aged 40–60 years drawn from six sites in four SSA countries [20, 21].

Five of the sites were INDEPTH-Network (International Network for the Demographic Evaluation of Populations and Their Health) Health and Demographic Surveillance Study (HDSS) sites namely Navrongo HDSS in Ghana, Nanoro HDSS in Burkina Faso, Nairobi urban HDSS in Kenya, and Agincourt and DIMAMO HDSS’s in South Africa. The sixth site was the South African MRC/University of the Witwatersrand Developmental Pathways to Health Research Unit (DPHRU) in Soweto, South Africa. Participants were randomly sampled from existing sampling frames in the various recruitment sites. Those who reported history of cardiovascular diseases (myocardial infarction, stroke) and chronic kidney disease were excluded from the analyses. Pregnant women, closely related individuals (first degree relatives), and those residents in the communities for less than 10 years were excluded.

### Independent variable: cardiovascular health index (CVHI)

The components of the CVHI as defined by AHA/ASA include blood pressure, fasting glucose, total cholesterol, body mass index, physical activity, diet, and smoking [8]. Each CVHI component was given a point score of 0, 1, or 2 to represent poor, intermediate, and ideal health respectively. Details of how the categories were defined are presented in Additional file 1: Table S1. The AHA recommended cutoffs are consistent with recommendations from the World Health Organization [22, 23] and other organizations [24]. These cutoffs have recently been used in a study in rural South Africa [19]. An overall CVHI score was computed as the sum of all 7 CVHI components, ranging from 0 to 14. We stratified participants into 3 mutually exclusive categories based on overall CVHI score: Ideal cardiovascular health (iCVH) defined as 12–14 points, intermediate cardiovascular health 8–11 points, and poor cardiovascular health 0–7 points [25]. Details of how the various components of CVHI were measured and defined in the AWI-Gen study have been published elsewhere [21].

Briefly, the history of smoking tobacco products was self-reported and defined as never smoked, previous smoker, and current smoker. Physical activity was measured as the level of moderate-to-vigorous physical activity (MVPA) from self-reported data on occupation, travel-related, and leisure time physical activity. Diet was measured as the self-reported daily number of servings of fruits and vegetables. Weight was measured to the nearest 0.1 kg using a digital 200 kg capacity scale (Kendon Medical, South Africa). Standing height to the nearest millimeter was measured using a Harpenden digital stadiometer (Holtain, Wales). The BMI was then calculated as weight divided by height squared in kilograms per square meter. Blood pressure was measured from the left arm of the participant, seated with the arm at the level of the heart and having rested 5–10 min. Three readings were taken with the first value discarded and an average of the second and third computed as blood pressure. Venous blood was taken with the participant having fasted overnight, and this sample was used to measure glucose and total cholesterol using colorimetric assays in a Randox Plus clinical chemistry analyzer (Crumlin, UK). The coefficient of variation of the laboratory measurements for glucose was 2.3% and 1.5% for total cholesterol. All analyses were performed in a centralized laboratory at the DPHRU in Soweto according to good laboratory practice and with external monitoring for quality control.

### Dependent variable: common carotid atherosclerosis

Carotid intima-media thickness was used as a marker of sub-clinical atherosclerosis. A carotid B-mode ultrasound scan (GE Healthcare, USA) with a 12 L-RS straight probe was used to measure the CIMT of the right and left common carotid artery (CCA). Details of the measurement procedure used in this population are described elsewhere [14, 21]. Briefly, the right and left intima-media thickness of the common carotid arteries (CCA) were measured at a single angle of 45 degree tilt with the participant lying supine and neck supported with a pillow. A mean of the right and left common CIMT (in micrometers) was computed as the outcome variable.

### Potential confounders and effect modifiers

Age was self-reported or calculated from reported date of birth while sex was reported at the time of recruitment. Household socio-economic status (SES) was determined based on household assets and quintiles calculated according to the practice implemented by the Demographic and Health Surveys (DHS) Program (http://www.dhsprogram.com/topics/wealth-index/Wealth-Index-Construction.cfm). A principal component analysis of the listed household assets is conducted and predicting factor scores generated from these analyses. The predicted factor scores are then categorized into quintiles for each of the participating sites separately [21]. Educational status was by asking the number years spent in school. Educational status was ascertained by asking the highest level of education (no formal education, primary, secondary or tertiary education). This was subsequently categorized as no formal education and yes formal education (primary, secondary and tertiary education). This took into consideration the number of years in each country at the different levels of school. HIV infection was self-reported in Burkina Faso and Ghana and testing was not offered due to the very low prevalence. Kenya and South Africa used locally available, government-approved rapid-test to conduct voluntary HIV rapid test. Antiretroviral therapy (ART) use was self-reported across all sites.

### Statistical analyses

All data analyses were conducted using STATA version 14.2 (Statacorp, College Station, TX, USA). We used descriptive and inferential statistics to summarize our data. Using Epanechnikov kernel function in STATA, density curves were plotted to determine the distribution of the CVHI score and common CIMT for the various countries represented in the AWI-Gen study (Additional file 2: Figs. S1 and S2). Continuous variables are reported as mean and standard deviation (± SD) due to approximate normal distribution and categorical variables as sample number with respective percentages. The differences in prevalence of overall cardiovascular health between women and men were examined using Pearson’s chi-squared test.

As the participants were resident in four different countries, we used multilevel mixed-effects generalized linear models to determine the associations of CVHI score with common CIMT. A variance co-variance method was used to obtain robust standard errors. Four progressively adjusted models were conducted as follows: model 1 was adjusted for age and sex; model 2 was adjusted for age, sex, level of education, and household socio-economic status; in model 3, we adjusted for all the model 2 variables in addition to HIV and ART use. The fixed effects portion of the model was considered to vary according to country and hence country was specified as the random effect part of the mixed effect model. Separate models were fitted for the combined populations, women and men and countries.

The country-specific models were fitted sequentially following the pattern explained above using adjusted linear regression models. Multilevel mixed-effects generalized linear models were conducted for South Africa which contained three different sites (site as the random effect). In the combined population, an individual participant data (IPD) meta-analyses was conducted. Pooled comparative sub-group analyses included comparison by country, sex, and household SES. Our sub-group analyses included household SES since it has previously been linked to poor health outcomes [26] and atherosclerosis [27]. Forest plots were subsequently plotted and weighted by sample size to avoid biased estimates (and for ease of comparison) and by default sample size instead of study weights appear on the plots. In addition, line plots of the predicted linear association between CVHI score and CIMT were drawn and presented in supplementary material (Additional file 2: Figs. S3 and S4). To determine the discriminatory power of each CVHI component metric to predict common CIMT, we generated dummy variables for the 3 levels of each CVHI metric using “poor” as the reference. We then conducted adjusted multilevel mixed effects linear regression analyses of the dummy variables, with adjustment for age, sex, educational status, and household SES in the pooled sample and for women and men separately. A two-tailed *p* < 0.05 was considered statistically significant in all inferential analyses.

## Results

After excluding participants (7.5%) with missing data, 9011 adults from Burkina Faso, Ghana, Kenya, and South Africa were included in these analyses. Basic characteristics are presented in Table 1. Fifty one percent of the study population was women and the average age was 51 ± 7 years. Physical activity levels were high (higher mean moderate-to-vigorous physical activity − minutes/week) in the populations from the two rural sites of West Africa. The average fruit and vegetable intake in the combined AWI-Gen population was 3 ± 2 servings per day. Mean BMI and systolic and diastolic blood pressure were highest in South Africa and Kenya and lowest in Ghana and Burkina Faso (Table 1).

**Table 1:** Socio-demographic characteristics of the AWI-Gen participants per research site

Characteristics	Total population ***N*** = 9011	Burkina Faso ***n*** = 2074	Ghana ***n*** = 1729	Kenya ***n*** = 1913	South Africa ***n*** = 3295
Women	4948 (50.8)	1035 (49.6)	936 (54.1)	1067 (54.1)	1910 (48.2)
Age in years	50.9 ± 6.9	49.8 ± 5.9	51.1 ± 5.8	48.8 ± 5.8	52.5 ± 8.0
Formal education in yes/no	6675 (63.8)	353 (17.0)	594 (29.6)	1795 (92.4)	3933 (88.5)
*Household SES	10.1 ± 4.1	11.3 ± 3.9	9.50 ± 4.6	11.1 ± 3.3	9.1 ± 4.0
**Household SES in quintiles**					
Q1 (poorest)	336 (16.2)	372 (18.5)	235 (12.1)	605 (13.4)	1548 (14.7)
Q2 (poorer)	404 (19.4)	360 (17.9)	432 (22.3)	1100 (24.4)	2296 (21.8)
Q3 (poor)	405 (19.5)	387 (19.2)	450 (23.2)	655 (14.6)	1897 (18.0)
Q4 (less poor)	386 (18.6)	473 (23.5)	396 (20.4)	949 (21.1)	2204 (20.9)
Q5 (least poor)	546 (26.3)	421 (20.9)	429 (22.1)	1194 (26.5)	2590 (24.6)
MVPA in minutes/weeks	1573 ± 1550	2077 ± 1590	2036 ± 1707	1749 ± 1732	1074 ± 1189
Servings of fruits and vegetables/day	2.9 ± 2.0	1.8 ± 0.9	3.4 ± 1.5	4.11 ± 2.5	2.23 ± 1.7
BMI in kg/m^2^	25.0 ± 6.8	20.9 ± 3.4	21.60 ± 3.6	25.40 ± 5.8	28.22 ± 7.6
SBP in mmHg	124.6 ± 21.8	115.6 ± 18.2	123.9 ± 21.6	120.0 ± 21.2	130.9 ± 21.7
DBP in mmHg	79.7 ± 3.7	73.5 ± 10.6	76.9 ± 12.7	78.4 ± 12.9	84.29 ± 14.2
Glucose in mg/dL	91.3 ± 29.4	90.9 ± 22.5	81.8 ± 13.21	97.8 ± 36.1	92.5 ± 33.5
Total cholesterol in mg/dL	148 ± 43.7	132 ± 36.9	125 ± 35.8	169 ± 41.9	159 ± 43.2
CVHI (0–14)	10.4 ± 2.0	10.8 ± 1.5	11.4 ± 1.6	10.7 ± 1.9	9.3 ± 2.0
Common CIMT in μm	637 ± 117	664 ± 118	690 ± 119	588 ± 104	620 ± 106

Values are *n* (%) or mean ± SD (standard deviation); *CVHI* cardiovascular health index, *CIMT* carotid intima-media thickness, *BMI* body mass index, *SBP* systolic blood pressure, *DBP* diastolic blood pressure, *SES* socio-economic status, *Q1 to Q5* wealth quintiles, *MVPA* moderate-to-vigorous physical activity in minutes per week. Formal education is defined as having attained primary or secondary or tertiary education; *Household SES presented as a mean ± SD of household assets

**Fig. 1:**
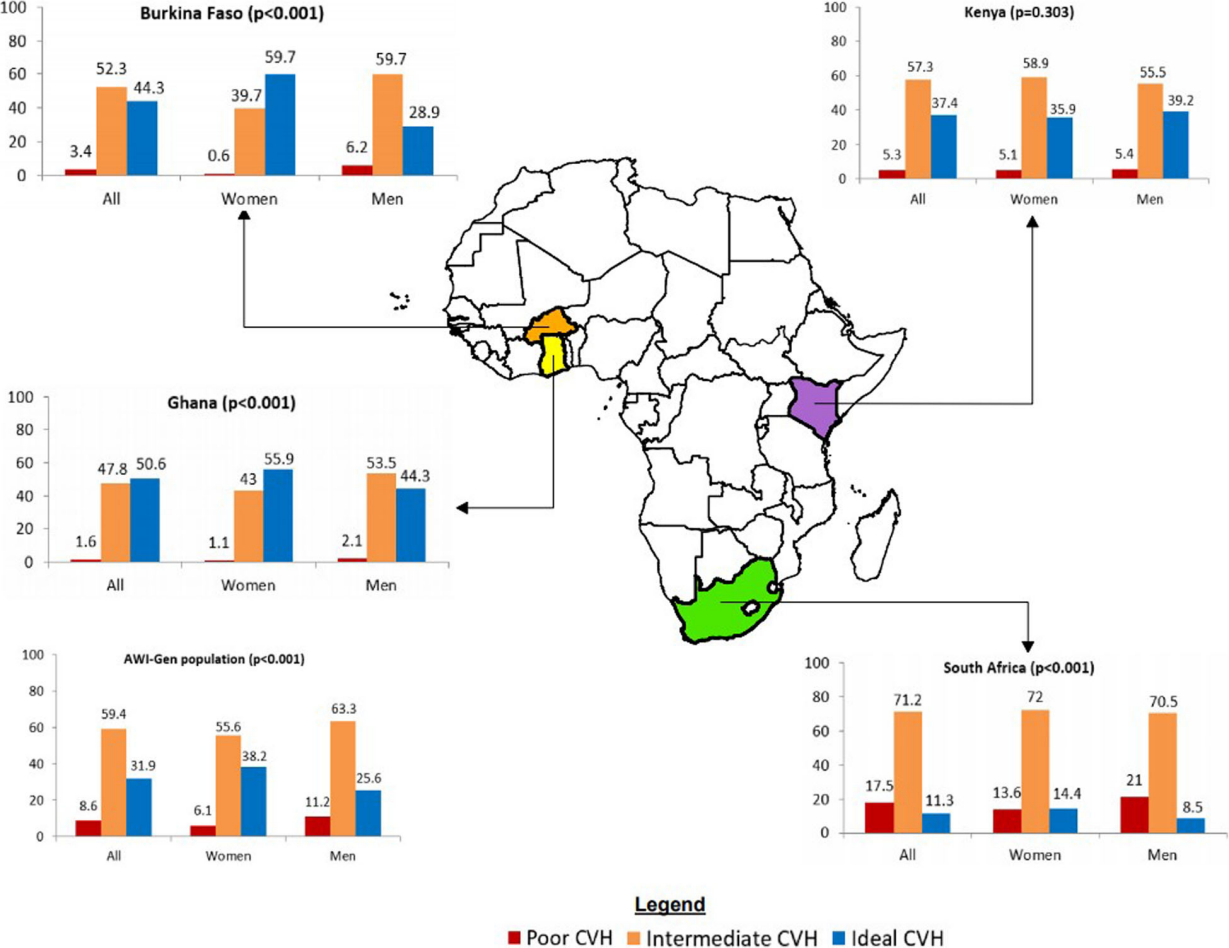
Map of Africa showing the prevalence of levels of cardiovascular health (CVH) for the various countries of the AWI-Gen study and according to sex, AWI-Gen population is the combined participant analysis. *p* values denote differences between women and men

The prevalence of each of the 3 levels of cardiovascular health (poor, intermediate, and ideal) for the total sample, the total sample stratified by sex, and the various countries is presented in Fig. 1. In the combined sample, iCVH was 31.9% with women (38.2%) having a higher prevalence than men (25.6%). Rural sites in Ghana (50.6%) and Burkina Faso (44.3%) had the highest prevalence of iCVH while the more urban sites in Kenya (37.4%) and South Africa (11.3%) had the lowest. The prevalence of the poor, intermediate, and ideal levels of each of the 7 CVHI components, according to sex and country, is presented in Table 2. The majority of participants were in the ideal category for total cholesterol, fasting glucose, physical activity, BMI, and blood pressure with unhealthy diet and smoking placing the majority of participants in the poor category. Burkina Faso and Ghana had more participants in the ideal category for most CVHI components than South Africa and Kenya, except diet and physical activity where Burkina Faso had the least ideal levels compared to other countries.

**Table 2:** The distribution of the poor, intermediate, and ideal levels of cardiovascular health for each of the 7 CVHI metrics in the AWI-Gen sample stratified by sex and country

CVHI metrics	Levels	Combined sample	Women	Men	Burkina Faso	Ghana	Kenya	South Africa
Smoking	Poor	984 (10.9)	103 (2.3)	881 (19.7)	124 (6.0)	190 (11.0)	309 (16.2)	361 (11.0)
	Intermediate	1600 (17.8)	73 (1.6)	1527 (34.1)	142 (6.8)	355 (20.5)	230 (12.0)	873 (26.5)
	Ideal	6427 (71.3)	4361 (96.1)	2066 (46.2)	1808 (87.2)	1184 (68.5)	1374 (71.8)	2061 (62.5)
Physical activity	Poor	1355 (15.0)	603 (13.3)	752 (16.8)	402 (19.4)	257 (14.9)	130 (6.8)	566 (17.2)
	Intermediate	442 (4.9)	227 (5.0)	215 (4.8)	63 (3.0)	86 (5.0)	129 (6.7)	164 (5.0)
	Ideal	7214 (80.1)	3707 (81.7)	3507 (78.4)	1609 (77.6)	1386 (80.2)	1654 (86.5)	2565 (77.8)
Healthy diet score	Poor	4266 (56.3)	2359 (56.2)	1907 (56.5)	1324 (82.4)	518 (30.0)	578 (30.2)	1846 (79.4)
	Intermediate	1946 (25.7)	1070 (25.5)	876 (26.0)	271 (16.9)	778 (45.0)	620 (32.4)	277 (11.9)
	Ideal	1361 (18.0)	770 (18.3)	591 (17.5)	12 (0.7)	433 (25.0)	715 (37.4)	201 (8.6)
Body mass index	Poor	1393 (15.5)	1092 (24.1)	301 (6.7)	36 (1.7)	45 (2.6)	375 (19.6)	937 (28.4)
	Intermediate	1673 (18.6)	914 (20.2)	759 (17.0)	177 (8.5)	168 (9.7)	494 (25.8)	834 (25.3)
	Ideal	5943 (66.0)	2529 (55.8)	3414 (76.3)	1861 (89.7)	1516 (87.7)	1043 (54.6)	1523 (46.2)
Blood pressure	Poor	1211 (13.4)	548 (12.1)	663 (14.8)	123 (5.9)	210 (12.1)	216 (11.3)	662 (20.1)
	Intermediate	2204 (24.5)	1006 (22.2)	1198 (26.8)	331 (16.0)	380 (22.0)	445 (23.3)	1048 (31.8)
	Ideal	5596 (62.1)	2983 (65.7)	2613 (58.4)	1620 (78.1)	1139 (65.9)	1252 (65.4)	1585 (48.1)
Fasting glucose	Poor	513 (5.7)	281 (6.2)	232 (5.2)	77 (3.7)	70 (4.0)	127 (6.6)	239 (7.3)
	Intermediate	1203 (13.4)	569 (12.5)	634 (14.2)	305 (14.7)	113 (6.5)	354 (18.5)	431 (13.1)
	Ideal	7295 (81.0)	3687 (81.3)	3608 (80.6)	1692 (81.6)	1546 (89.4)	1432 (74.9)	2625 (79.7)
Total cholesterol	Poor	350 (3.9)	185 (4.1)	165 (3.7)	44 (2.1)	10 (0.6)	107 (5.6)	189 (5.7)
	Intermediate	805 (8.9)	425 (9.4)	382 (8.5)	97 (4.7)	44 (2.5)	282 (14.6)	382 (11.6)
	Ideal	7856 (87.2)	3927 (86.6)	3929 (87.8)	1933 (93.2)	1675 (96.9)	1524 (79.7)	2724 (82.7)

Data presented as *n* (%); levels of CVH = cardiovascular health which is defined as poor (CVHI score of 0–7), intermediate (CVHI score of 8–11), and optimum (CVHI score of 12–14)

**Fig. 2:**
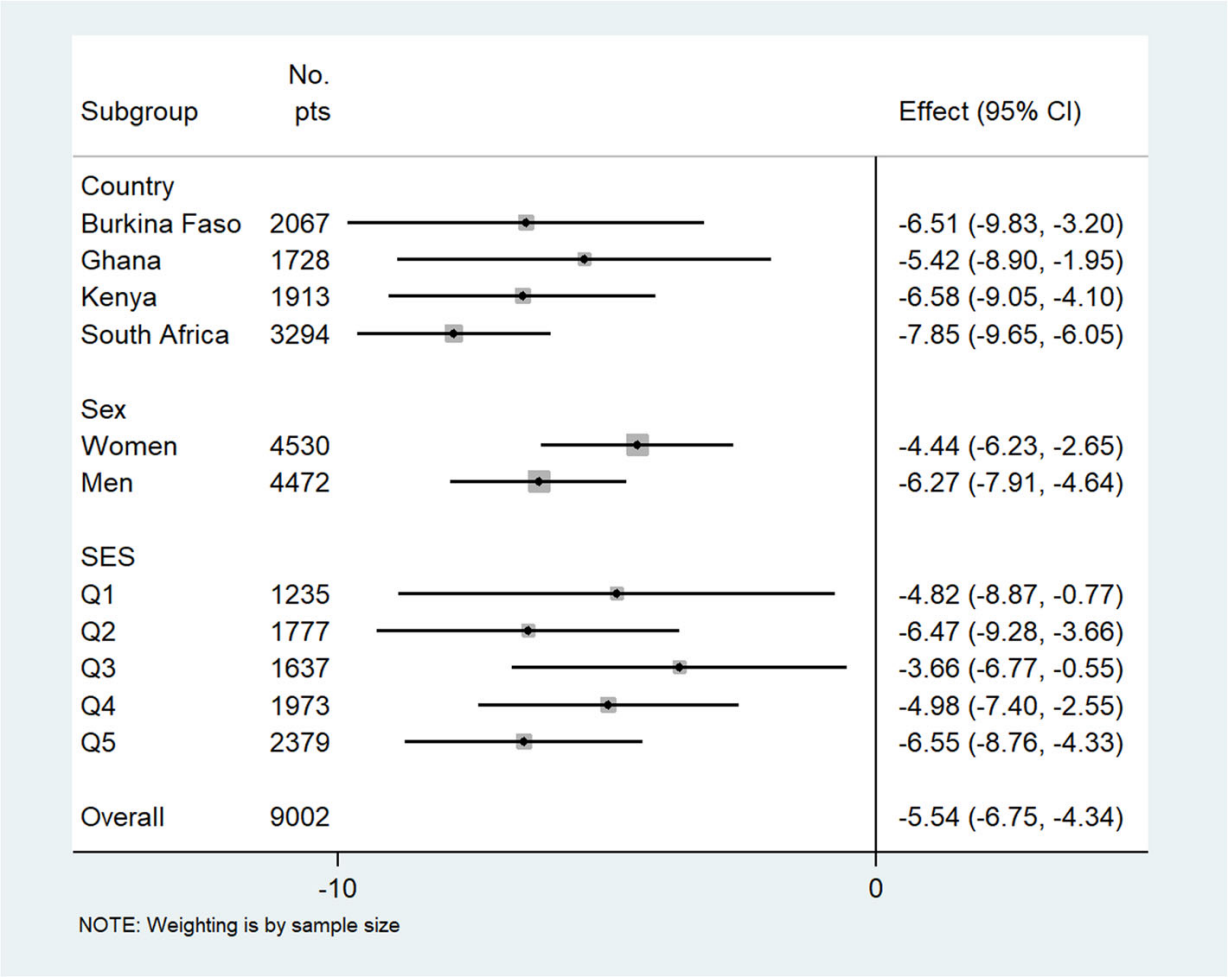
Adjusted association of cardiovascular health index (CVHI) score with common carotid intima-media thickness (CIMT in μm) in the combined AWI-Gen population stratified by country, sex, and socio-economic status (SES) subgroups. Effect is represented by β-coefficients and is a change in mean CIMT (in micrometers) with each unit increase in the CVHI score (0–14). Wealth quintile defined as Q1 = poorest, Q2 = poorer, Q3 = poor, Q4 less poor, and Q5 = least poor. Adjusted for age, educational status, household SES, HIV + ART, self-reported history of stroke, heart attack, congestive heart failure, and chronic kidney disease

**Fig. 3:**
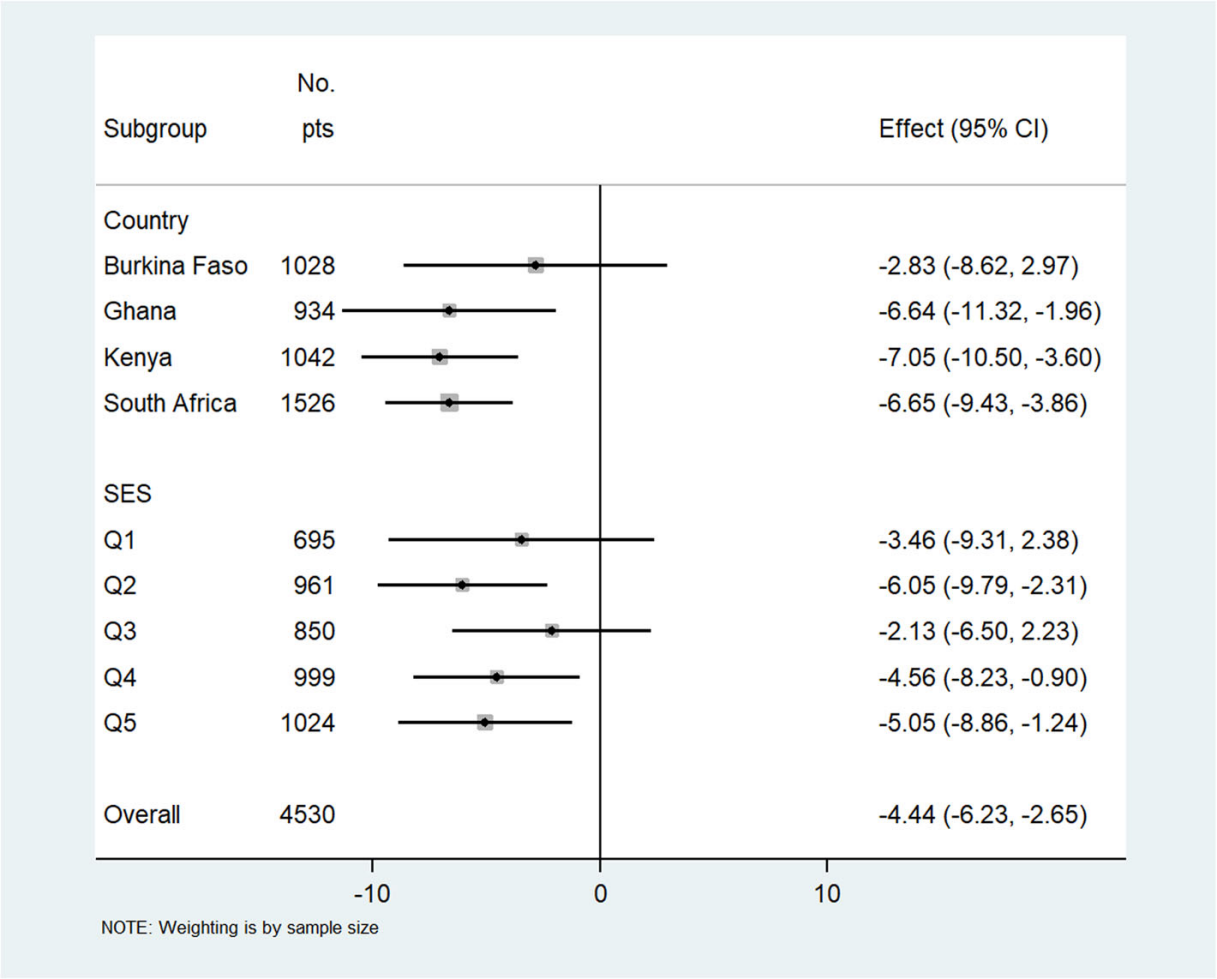
Adjusted association of cardiovascular health index (CVHI) score with common carotid intima-media thickness (CIMT in μm) in women stratified by country and socio-economic status (SES). Effect is represented by β-coefficients and is a change in mean CIMT (in micrometers) with each unit increase in the CVHI score (0–14). Wealth quintile defined as Q1 = poorest, Q2 = poorer, Q3 = poor, Q4 less poor, and Q5 = least poor. Adjusted for age, educational status, household SES, HIV + ART, self-reported history of stroke, heart attack, congestive heart failure, and chronic kidney disease

**Fig. 4:**
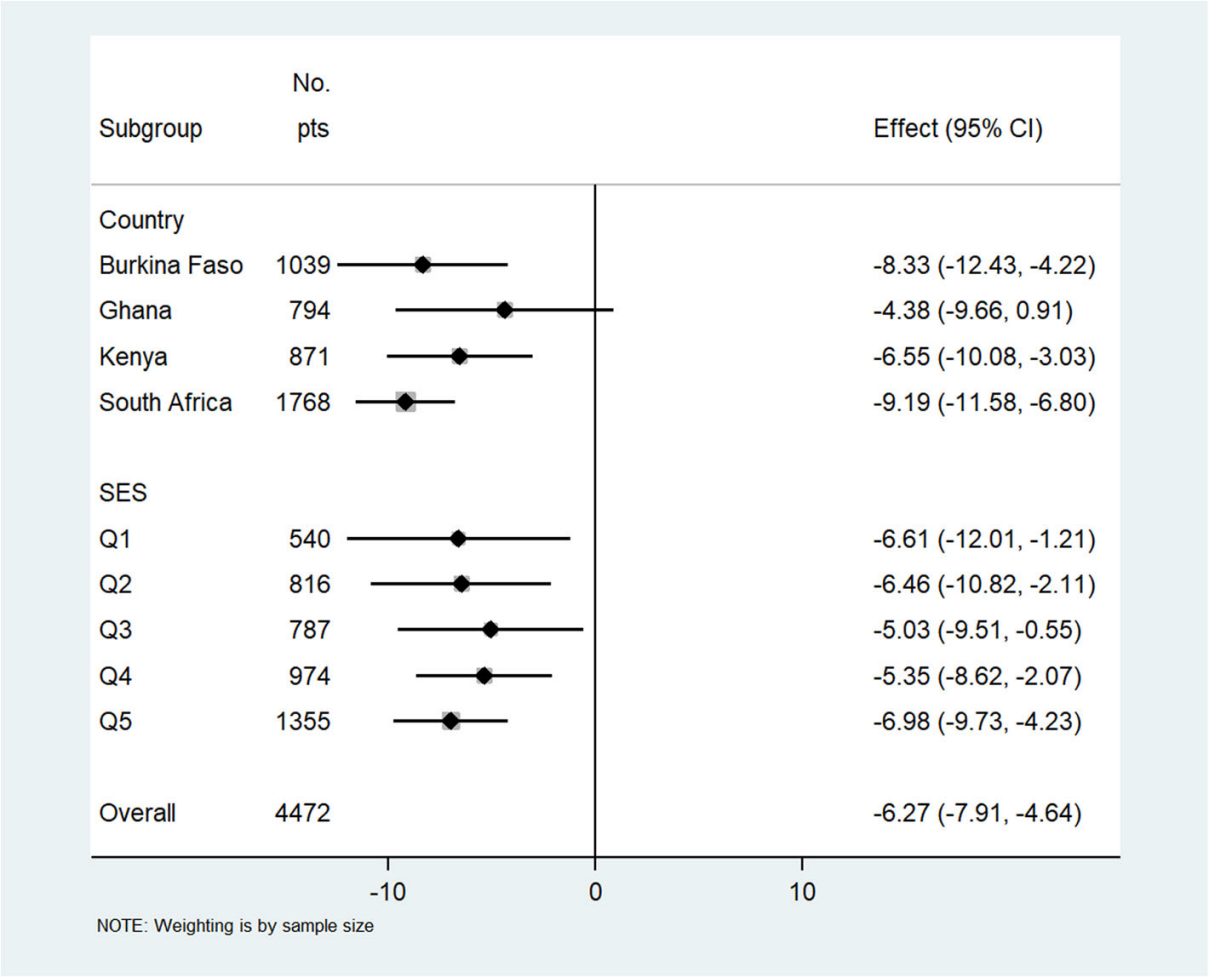
Adjusted association of cardiovascular health index (CVHI) score with common carotid intima-media thickness (CIMT in micrometers) in men stratified by country and socio-economic status (SES). Effect is represented by β-coefficients and is a change in mean CIMT (in μm) with each unit increase in the CVHI score (0–14). Wealth quintile defined as Q1 = poorest, Q2 = poorer, Q3 = poor, Q4 less poor, and Q5 = least poor. Adjusted for age, educational status, household SES, HIV + ART, self-reported history of stroke, heart attack, congestive heart failure, and chronic kidney disease

There were inverse linear associations between CIMT and CVHI in our combined population (*r* = − 0.064; *p* < 0.001) and for Burkina Faso (*r* = − 0.126; *p* < 0.001), Ghana (*r* = − 0.085; *p* < 0.001), Kenya (*r* = − 0.178; *p* < 0.001), and South Africa (*r* = − 0.199; *p* < 0.001), and these are presented as line plots in Additional file 2: Figs. S3 and S4. In the adjusted multilevel mixed-effects generalized linear regression model for the combined population, a unit increase in the CVHI was associated with − 5.54 [− 6.75, − 4.32] μm decrease in common CIMT (Fig. 2). The risk of atherosclerosis (high common CIMT) was progressively lower with increasing CVHI in each country, sex, and household SES category in both the combined sample (Fig. 2) and the sex stratified analyses (Figs. 3 and 4). A similar inverse association was observed in women (− 4.44 [− 6.23, − 2.65]) and in men (− 6.27 [− 7.98, − 4.64]) (Figs. 3 and 4, respectively).

Sequential adjustments of the regression models are presented in Table 3 and demonstrate minimal effects of adjustment on the β-coefficients. All models demonstrated a significant inverse association between the CVHI and common CIMT, except in women from Burkina Faso and men from Ghana, where the associations were again negative but with the upper 95% CI just overlapping zero. In adjusted analyses looking at the contribution of the individual components of CVHI to common CIMT, we observed that blood pressure, BMI, and hyperglycemia were associated with common CIMT in the combined population, while smoking and physical activity were associated with common CIMT in women only (see Table 4).

**Table 3:** Association of CVHI score with common CIMT (μm) in women and men in the combined AWI-Gen sample and by countries

	All countries	Burkina Faso	Ghana	Kenya	South Africa
	*β* [95% CI]	*β* [95% CI]	*β* [95% CI]	*β* [95% CI]	*β* [95% CI]
**Combined sample**					
Model 1	− 7.39 [−8.49, −6.27]	− 6.48 [− 9.80, − 3.16]	− 5.42 [− 8.89, − 1.94]	− 6.79 [− 9.24, − 4.35]	− 8.31 [− 10.1, − 6.52]
Model 2	− 7.15 [− 7.89, − 6.41]	− 6.43 [− 9.76, − 3.09]	− 5.46 [− 8.94, − 1.97]	− 6.66 [− 9.12, − 4.19]	− 7.66 [− 9.47, − 5.85]
Model 3	− 7.22 [− 8.04, − 6.39]	− 6.51 [− 9.83, − 3.19]	− 5.42 [− 8.91, − 1.94]	− 6.57 [− 9.05, − 4.10]	−7.85 [− 9.65, − 6.05]
Model 4	− 7.11 [− 7.74, − 6.48]	− 6.42 [− 9.75, − 3.09]	− 5.51 [− 8.99, − 2.02]	− 6.63 [− 9.10, − 4.16]	− 7.49 [− 9.31, − 5.68]
**Women**					
Model 1	− 5.98 [− 7.44, − 4.52]	− 2.05 [− 7.73, 3.62]	− 6.04 [− 10.60, − 1.46]	− 6.81 [− 10.2, − 3.43]	− 6.89 [− 9.69, − 4.10]
Model 2	− 5.85 [− 7.00, − 4.71]	− 2.18 [− 7.98, 3.62]	− 6.59 [− 11.20, − 1.96]	− 7.05 [− 10.50, − 3.64]	− 6.62 [− 9.39, − 3.86]
Model 3	− 4.87 [− 5.69, − 4.05]	− 2.83 [− 8.63, 2.97]	− 6.64 [− 11.30, − 1.96]	− 7.05 [− 10.50, − 3.59]	− 6.65 [− 9.44, − 3.86]
Model 4	− 5.93 [− 7.02, − 4.85]	− 2.29 [− 8.10, 3.53]	− 6.79 [− 11.40, − 2.15]	− 7.16 [− 10.60, − 3.71]	− 6.52 [− 9.34, − 3.69]
**Men**					
Model 1	− 7.83 [− 9.79, − 5.86]	− 8.62 [− 12.7, − 4.52]	− 4.69 [− 10.1, 0.66]	− 6.66 [− 10.20, − 3.15]	− 9.36 [− 11.70, − 7.01]
Model 2	− 7.42 [− 9.21, − 5.63]	− 8.25 [− 12.4, − 4.14]	− 4.56 [− 9.91, 0.79]	− 6.51 [− 10.00, − 2.99]	− 8.84 [− 11.20, − 6.44]
Model 3	− 5.68 [− 6.57, − 4.80]	− 8.33 [− 12.4, − 4.22]	− 4.38 [− 9.67, 0.92]	− 6.55 [− 10.10, − 3.02]	− 9.19 [− 11.60, − 6.80]
Model 4	− 7.38 [− 9.11, − 5.66]	− 8.18 [− 12.3, − 4.07]	− 4.47 [− 9.81, 0.87]	− 6.42 [− 9.93, − 2.91]	− 8.71 [− 11.10, − 6.32]

Effect measured as β-coefficient (change in mean CIMT in μm per unit change in CVHI score); model 1 adjusted for age and sex; model 2 adjusted for age, sex, education status, household SES, and site; model 3 adjusted for self-reported history of stroke, heart attack, congestive heart failure, and chronic kidney disease in addition to model 2; model 4 was adjusted for model 2 plus chronic kidney disease and HIV plus antiretroviral therapy (ART) use; model 1 for women and men were adjusted for only age. *CVHI* cardiovascular health index, *CIMT* carotid intima-media thickness

**Table 4:** Adjusted association of components of the CVHI metric with common CIMT in the AWI-Gen population stratified by sex

CVHI metrics		All countries		Women		Men	
	Levels	β [95%CI]	***p*** value	β [95% CI]	***p*** value	β[95% CI]	***p*** value
Smoking	Poor	Ref	Ref	Ref	Ref	Ref	Ref
	Intermediate	− 1.01 [− 13.7, 11.7]	0.083	− 11.4 [− 23.9, 1.15]	< 0.001	1.41 [− 12.9, 15.7]	0.658
	Ideal	3.28 [− 0.09, 7.35]		− 7.04 [− 10.1, − 3.98]		3.83 [− 16.7, 9.04]	
Physical activity	Poor	Ref	Ref	Ref	Ref	Ref	Ref
	Intermediate	− 17.5 [− 34.9, − 0.08]	0.078	− 19.8 [− 34.0, − 5.59]	< 0.001	− 10.8 [− 34.2, 12.6]	0.365
	Ideal	− 12.8 [− 24.8,-0,78]		− 9.28 [− 14.1, − 4.51]		− 12.63 [− 30.4, 5.14]	
Diet	Poor	Ref	Ref	Ref	Ref	Ref	Ref
	Intermediate	1.92 [− 4.24, 8.09]	0.169	5.68 [− 1.91, 13.3]	0.059	− 2.47 [− 13.3, 8.33]	0.654
	Ideal	6.75 [− 0.24, 13.7]		7.55 [− 1.08, 16.2]		6.11 [0.32, 11.9]	
Body mass index	Poor	Ref	Ref	Ref	Ref	Ref	Ref
	Intermediate	− 4.06 [− 17.4, − 2.96]	< 0.001	− 13.2 [− 25.5, − 0.94]	< 0.001	2.57 [− 20.4, 25.6]	< 0.001
	Ideal	− 28.2 [− 44.2, − 12.2]		− 33.6 [− 50.6, − 16.6]		− 32.7 [− 55.1, − 10.4]	
Hypertension	Poor	Ref	Ref	Ref	Ref	Ref	Ref
	Intermediate	− 17.7 [− 26.0, − 9.28]	< 0.001	− 7.30 [− 20.4, − 5.75]	< 0.001	− 23.8 [− 27.0, − 20.6]	< 0.001
	Ideal	− 31.4 [− 42.4, − 20.3]		− 20.2 [− 39.1, − 1.28]		− 28.9 [− 38.1, − 19.7]	
Fasting glucose	Poor	Ref	Ref	Ref	Ref	Ref	Ref
	Intermediate	− 18.2 [− 35.8, − 0.68]	< 0.001	− 23.3 [− 36.8, − 9.86]	< 0.001	−8.79 [− 38.7, 21.1]	0.377
	Ideal	− 14.2 [− 32.8, − 4.42]		− 21.8 [− 35.2, − 8.27]		− 0.57 [− 29.1, 27.9]	
Total cholesterol	Poor	Ref	Ref	Ref	Ref	Ref	Ref
	Intermediate	− 1.89 [− 28.3, 24.5]	0.958	5.45 [− 15.1, 25.9]	0.542	− 15.8 [− 43.3, 11.6]	0.265
	Ideal	− 0.58 [− 28.6, 27.5]		10.53 [− 11.2, 32.2]		− 17.8 [− 41.9, 6.21]	

Adjusted for age, sex, educational status, and socio-economic status adjusted for in the combined population while age, educational status, and socio-economic status adjusted for in the sex stratified analyses. Effect is represented by β-coefficients and is a change in mean CIMT (in μm) with each unit increase in the CVHI components. *CVHI* cardiovascular heath index

## Discussion

Results from this large population-based study of middle-aged men and women from four countries in sub-Saharan Africa highlight relatively high levels of ideal cardiovascular health. We have also shown a significant inverse linear association between increasing CVH score and common CIMT independent of age, educational status, household socio-economic status, HIV infection, and ART use and prior history of cardiovascular or chronic kidney diseases. To our knowledge, this study is the largest to demonstrate an association between the CVHI metric and subclinical atherosclerosis in an apparently healthy adult population from multiple countries in SSA.

The prevalence of ideal cardiovascular health (iCVH) was 31.9% in the combined AWI-Gen population, and this was higher than the 6.8% reported in the multi-ethnic study of atherosclerosis (MESA) conducted among Caucasians, Chinese, African Americans, and Hispanics in the USA [25]. Similarly, data from the Behavioral Risk Factor Surveillance System study which used information obtained telephonically from nearly 1.4 million subjects in the USA reported a much lower prevalence of iCVH (5.1%) in 2009 than reported in the current study [28]. The iCVH levels among our participants from Ghana was 50.9% and higher than reported by the Research on Obesity and Diabetes among African Migrants (RODAM) study conducted among Ghanaians residing in rural (25.7%) and urban (7.5%) areas and their counterparts in the diaspora (< 5%) [29]. Further to this, a systematic review by Peng et al. showed that participants from Africa were likely to have higher proportions of ideal CVH compared to North and South Americans, Asians, Europeans, and the Oceania’s [30]. The RODAM study, however, used the comprehensive five dietary components (as described in the original CVHI) to define healthy diet whereas only two components were available for use in the AWI-Gen study. This may therefore result in over classifying AWI-Gen participants into the ideal diet category. This notwithstanding, the broad range of iCVH levels observed in the various settings may be a reflection of the level of ongoing epidemiological transition from communicable to non-communicable diseases. Thus, the iCVH levels were much higher in rural communities in Ghana and Burkina Faso and lower in the more urban settings of Kenya and South Africa and lowest in Europe and the USA.

We observed an inverse association of the iCVH with common CIMT levels independent of age, educational status, socio-economic status, HIV infection, and ART use. Similar to our findings, several other studies conducted in Caucasian, African-American, and Asian populations have observed increasing CVHI score is associated with lower risk of subclinical atherosclerosis [15, 17, 31–35]. It has been reported that a proportion of residual phenotypic variance is due to the additive effects of genes [36]. Although we did not adjust for the effect of genes in this paper, the Emory Twin Study did report that shared genetic and other familial factors do not confound the association of CVHI with CIMT [15]. Our results did not show any significant disparities in the association between CVHI and CIMT across different levels of socio-economic wealth quintiles as has been previously reported in a multi-ethnic population, including African Americans, in the USA [25]. This may be due to the fact that participants in our population did not show significant difference in wealth distribution.

The rising burden of CVD morbidity and mortality in SSA has been reported to occur in younger age groups [1, 37]. This, coupled with high levels of unawareness of risk factors and challenges with secondary prevention, calls for the evaluation of simple tools that can be used in routine screening for subjects with poor CVH in primary prevention efforts. The significant independent association of CIMT with the CVHI demonstrated in this study makes a strong case for the utility of the CVHI metric in the routine screening of CVD risk in the broader SSA population irrespective of SES status.

We observed in the combined sample that BMI, blood pressure, and hyperglycemia were the CVHI metrics most strongly associated with common CIMT. The former 2 components were also associated with common CIMT in both women and men while physical activity and smoking related with common CIMT in women but not men. This could be due to the fact that women were less likely to smoke compared to men and equally had healthier physical activity levels than men. These findings have implications for prioritizing particular CVHI components for the primary prevention of atherosclerotic cardiovascular diseases in SSA. Although total cholesterol did not correlate with CVHI, cholesterol levels in Africans remain lower than in the Western World. However, with urbanization, cholesterol levels are increasing and thus may well contribute in the near future, to atherosclerotic cardiovascular disease in SSA [38].

Our study does have some limitations. Reliance on self-reported smoking, physical activity, HIV infection status, and ART use may result in recall and social desirability biases. Diet was ascertained as self-reported servings of fruits and vegetables per day instead of derived from five components (fruits and vegetables per day; portions of fish a week; servings of fiber-rich whole grains per day; sodium per day and sugar-sweetened beverages per week) as recommended by AHA, which could have resulted in misclassification of certain participants in terms of their dietary intake. In addition, the cut points used for classifying each of the components of the CVHI metric were largely based on those used in populations of European ancestry. This brings into question whether these cut points are appropriate for use in African populations. However, the cut points used in the CVHI for defining cholesterol, blood pressure, glucose, and BMI levels are used globally for the categorization of these disease markers and are recognized cut points in the clinical guidelines of many African nations; their use within these countries has not been widely questioned. Other cut points have been challenged for their use within African populations and include those for HDL and triglycerides [39], HbA1c [40], and waist circumference [41], but none of these factors are used within the CVHI metric.

Another possible shortcoming of the use of the CVHI in African populations is the high prevalence of underweight which is classified in the ideal category for BMI but is associated with increased cardiovascular risk. However, a large meta-analysis has shown that low BMI is more strongly associated with respiratory disease than CVD mortality, but it must be noted that this review included few studies from Africa [42]. Future studies confirming the applicability of the CVHI criteria to African populations, especially those for BMI, would be important. In the current study, it was essential to use the existing CVHI criteria so that we were able to compare our data to those from studies conducted in both African and non-African populations.

Despite these limitations, the greatest strength of this work is that it used a large cohort of African individuals from 4 different SSA countries and a standardized data collection protocol, with laboratory assays performed at a single laboratory. In addition, a large number of possible confounding or modulatory variables were measured for use in the multiple regression models. Furthermore, this is the largest African study to use the CVHI to assess cardiovascular health and its association with common CIMT and provides important information on the utility of this metric for screening for individuals at high risk for CVD in resource-poor environments.

## Conclusion

The CVHI score has a significant inverse, independent association with common CIMT in older adults across four sub-Saharan African countries. This makes a case for its use as a simple tool to monitor cardiovascular health at a population and clinical level in SSA. The current study demonstrates that primary prevention of atherosclerotic CVDs in SSA should focus on reducing smoking and obesity, improved control of hypertension and hyperglycemia, and higher levels of physical activity.

## Supplementary Information

**Additional file 1: Table S1.** Scoring and definition of components of Cardiovascular Health Index.

**Additional file 2: Figure S1.** Kernel density of mean distribution number of ideal cardiovascular health metrics (0–14) by AWI-Gen study countries. **Figure S2.** Kernel density of mean CIMT distribution in μm by AWI-Gen study countries. **Figure S3.** The linear association between CVH score and CIMT in the combined AWI-Gen population. **Figure S4.** The linear association between CVH score and CIMT in the combined four participating countries of the AWI-Gen study.

## Data Availability

The datasets generated and/or analyzed during the current study will be made publicly available in the European Genome-phenome Archive under the set of projects related to the Human Heredity and Health in Africa (H3Africa) Consortium. Details concerning access to data and DNA can be found in the document titled H3Africa Data and Biospecimen Access Committee Guidelines, available in the consortium documents section of the H3Africa website (www.h3africa.com).

## Abbreviations

AHA/ASA: American Heart Association and the American Stroke Association
AWI-Gen: African Wits-INDEPTH Genomic studies
BMI: Body mass index
CVD: Cardiovascular disease
CIMT: Carotid-Intima media thickness
CVHI: Cardiovascular Health Index
HDSS: Health and Socio-demographic Surveillance System
SSA: Sub-Saharan Africa
