# Supplementary material for: Poor cardiovascular health is associated with subclinical atherosclerosis in apparently healthy sub-Saharan African populations: an H3Africa AWI-Gen study
Source: BMC Med. 2021 Feb 10;19:30. doi: 10.1186/s12916-021-01909-6 (PMC7874493; doi:10.1186/s12916-021-01909-6)
Supplement: Supplementary file 1 — Additional file 1: Table S1. Scoring and definition of components of Cardiovascular Health Index. [file 12916_2021_1909_MOESM1_ESM.docx]

**Table S1:** Scoring and definition of components of Cardiovascular Health Index

| **CVHI metric** | **Levels** | **Score** | **Definition** |
| --- | --- | --- | --- |
| Smoking* | Poor | 0 | Current |
|  | Intermediate | 1 | Former |
|  | Ideal | 2 | Never |
| Physical activity | Poor | 0 | No physical activity |
|  | Intermediate | 1 | 1-149mins/week of MVPA^†^ |
|  | Ideal | 2 | ≥150mins/week of MVPA^†^ |
| Healthy diet‡ score | Poor | 0 | < 2 servings per day |
|  | Intermediate | 1 | 2 to 4 servings per day |
|  | Ideal | 2 | ≥ 5 servings per day |
| Body mass index | Poor | 0 | ≥30 kg/m^2^ |
|  | Intermediate | 1 | 25 to 29.99 kg/m^2^ |
|  | Ideal | 2 | <25 kg/m^2^ |
| Blood pressure | Poor | 0 | SBP ≥140 or DBP ≥90 mm Hg |
|  | Intermediate | 1 | SBP 120-139 or DBP 80-89 mm Hg or treated with antihypertensive to normal level |
|  | Ideal | 2 | SBP<120 or DBP<80 mm Hg, without antihypertensive medication |
| Fasting glucose | Poor | 0 | ≥126 mg/dL |
|  | Intermediate | 1 | 100 to 125 mg/dL or treated with anti-diabetes to normal level |
|  | Ideal | 2 | <100 mg/dL, without anti-diabetes medication |
| Total cholesterol | Poor | 0 | ≥240 mg/dL |
|  | Intermediate | 1 | 200 to 239 mg/dL or treated to <200 mg/dL |
|  | Ideal | 2 | <200 mg/dL, without lipid lowering medication |

*Self-reported smoking status was reported as never smoked, previous smoker and current smoker; there was no data on duration of smoking; **†**MVPA indicates moderate-to-vigorous physical activity; ‡Healthy diet score modified based on available data in the AWI-Gen study on the number of servings of fruits and vegetable per day.
