# Supplementary material for: Poor cardiovascular health is associated with subclinical atherosclerosis in apparently healthy sub-Saharan African populations: an H3Africa AWI-Gen study
Source: BMC Med. 2021 Feb 10;19:30. doi: 10.1186/s12916-021-01909-6 (PMC7874493; doi:10.1186/s12916-021-01909-6)
Supplement: Supplementary file 2 — Additional file 2: Figure S1. Kernel density of mean distribution number of ideal cardiovascular health metrics (0–14) by AWI-Gen study countries. Figure S2. Kernel density of mean CIMT distribution in μm by AWI-Gen study countries. Figure S3. The linear association between CVH score and CIMT in the combined AWI-Gen population. Figure S4. The linear association between CVH score and CIMT in the combined four participating countries of the AWI-Gen study. [file 12916_2021_1909_MOESM2_ESM.docx]

Figure S1: Kernel density of mean distribution number of ideal cardiovascular health metrics (0-14) by AWI-Gen study countries

Figure S2: Kernel density of mean CIMT distribution in µm by AWI-Gen study countries

Figure S3: The linear association between CVH score and CIMT in the combined AWI-Gen population

Figure S4: The linear association between CVH score and CIMT in the combined four participating countries of the AWI-Gen study
